# A Comparative Study Among Elite Female Volleyball Players and the General Population: Are All Valvular Regurgitations Benign?

**DOI:** 10.7759/cureus.84344

**Published:** 2025-05-18

**Authors:** Akin Torun, Seckin Sari, Arda Cinar, Turan Cetinkaya, Seher Yildiz, Rayhana El Mounjali, Batur Kanar

**Affiliations:** 1 Department of Cardiology, Sultan II. Abdulhamid Han Training and Research Hospital, Istanbul, TUR; 2 Department of Orthopedics and Traumatology, Istanbul Bilgi University, Istanbul, TUR; 3 Department of Orthopedics, SCA Clinic Istanbul, Istanbul, TUR; 4 Department of Physical Education and Sports Education, Faculty of Sports Sciences, Ahi Evran University, Kırsehir, TUR; 5 Faculty of Medicine, Bahcesehir University, Istanbul, TUR; 6 Department of Cardiology, Faculty of Medicine, Istinye University, Istanbul, TUR

**Keywords:** athlete's heart, echocardiography, mitral insufficiency, tricuspid regurgitation, volleyball players

## Abstract

Background: Despite the global popularity of volleyball as an Olympic sport, there is a relative paucity of research concerning its cardiovascular implications. Valvular regurgitation is a common finding among athletes and may lead to diagnostic ambiguity, particularly in elite female volleyball players.

Objectives: In this study, we conducted a comparative echocardiographic analysis between elite female volleyball athletes and a control group from the general population.

Method: Echocardiographic data of elite volleyball players from the Turkish Sultans League were compared with those of sedentary female controls. The frequency and severity of heart valve regurgitation were evaluated.

Results: A total of 31 elite athletes were included in the study and compared to 37 age- and sex-matched healthy controls. Echocardiographic measurements revealed that the left ventricular end-diastolic diameter was higher in athletes than in controls (48.0 ± 2.6 mm vs. 43.7 ± 2.5 mm, p = 0.04). Additionally, grade 2 mitral (p = 0.04) and tricuspid (p = 0.003) valve insufficiencies were more frequently observed in elite athletes compared to the control group. However, aortic valve insufficiency was not observed as a characteristic of the athlete's heart. All participants were asymptomatic.

Conclusion: Elite female volleyball players demonstrated a higher prevalence of mitral and tricuspid valve insufficiencies compared to healthy controls. Moreover, the left ventricular end-diastolic diameter was significantly larger in athletes. However, no significant differences were observed in other echocardiographic parameters, and aortic valve insufficiency was not identified as a characteristic of the athlete’s heart. Further studies are needed to provide more sport-specific data on these findings.

## Introduction

Exercise training induces a range of morphological and functional cardiac adaptations collectively referred to as the "athlete’s heart" [1]. This phenomenon has gained increasing importance with the growing participation in competitive sports and heightened societal focus on physical fitness. Given the established influence of socioeconomic status on cardiovascular outcomes in the general population, incorporating these factors into the interpretation of athlete’s heart is essential, as disparities in access to healthcare, nutrition, and training resources may affect both the presentation and stratification of cardiovascular risk among athletes [2]. As a result, distinguishing between physiological adaptations and potentially life-threatening cardiovascular pathologies is a central challenge in sports cardiology. Pathological conditions such as hypertrophic cardiomyopathy, dilated cardiomyopathy, Marfan syndrome, and arrhythmogenic right ventricular cardiomyopathy may exhibit phenotypic features that closely resemble those of the athlete’s heart [3]. Furthermore, the extent of exercise-induced changes often exceeds conventional clinical thresholds, underscoring the need for accurate differentiation between adaptation and disease [4]. Although detecting cardiac disease in athletes is critical, it is equally important to avoid misclassifying healthy individuals, as this may lead to undue anxiety or unjustified exclusion from athletic participation.

Echocardiography remains a key modality for assessing cardiac structure, function, and responses to training. A wide range of techniques, including M-mode, two-dimensional (2D) imaging, Doppler modalities, tissue Doppler imaging, color tissue Doppler, and speckle-tracking echocardiography, have been employed to characterize these adaptations [5]. The nature of cardiac remodeling varies based on training intensity, duration, and sport-specific demands [6]. Volleyball is characterized by intermittent high-intensity activity, frequent jumping, and short sprints, all of which impose both dynamic and static loads on the cardiovascular system. These unique demands may result in distinctive patterns of cardiac adaptation, thereby justifying the need for sport-specific evaluation.

Despite its status as a globally popular and Olympic-level sport, volleyball remains underrepresented in echocardiographic research. The current study specifically focuses on female volleyball players, a group that is particularly underexplored in the sports cardiology literature. This focus is justified not only by the scarcity of data but also by the existence of sex-related physiological differences in cardiac structure and response to training. Understanding these differences is essential for developing accurate diagnostic criteria and safe participation guidelines for female athletes.

One diagnostic ambiguity in athletic cardiac assessment is the observation of valvular regurgitation, particularly in asymptomatic individuals. Although often considered a benign finding in athletes, the clinical significance of such regurgitation remains uncertain. Its presence may reflect physiological remodeling due to training-related hemodynamic stress; however, in some cases, it could indicate early pathological changes or predispose individuals to future cardiovascular complications. Therefore, documenting the prevalence and severity of valve regurgitation in specific athlete populations warrants further investigation.

Previous studies have reported varying rates of valvular regurgitation among athletes, with mild mitral and tricuspid regurgitation frequently observed as part of the athlete’s heart spectrum. However, there is a paucity of data specifically addressing elite volleyball players, and even fewer studies focus on female athletes. This gap limits the development of evidence-based reference standards.

This study aims to evaluate and compare the prevalence and severity of valvular regurgitation, as well as structural cardiac parameters, in elite female volleyball players versus the general population. By doing so, the study aims to generate sport- and sex-specific echocardiographic data that can inform clinical practice and contribute to the broader field of sports cardiology.

## Materials and methods

Study population and data collection

Volleyball players playing in the Turkish Sultans League were included in this study. The participants are from six different nations, the majority of whom are players in their national teams, and six of whom are Olympians. Echocardiography data of volleyball players performed during their health checks at the beginning of the 2024-2025 season were analyzed. Female healthy volunteers aged between 18 and 40, with no chronic disease and no cardiac pathology detected in their examinations, who applied to the cardiology outpatient clinic between October and November, were consecutively included in the study as a control group. Participants in the control group who exercised more than four hours per week were excluded from the study. Informed consent was obtained from all participants. All echocardiographic examinations were evaluated by two experienced cardiologists. All athletes and the control group were asymptomatic. 

The echocardiography data of the two groups were compared. It was analyzed whether there was a difference in the frequency of heart valve regurgitation. No leakage was categorized as grade 0, trivial physiological leakage as grade 1, and mild leakage as grade 2. It was categorized as grade 2 with the criteria of effective regurgitant orifice area (EROA) of 0.2-0.29 cm^2^ and regurgitant volume of 30-44 ml [7]. Grade 0 refers to the absence of any valve regurgitation. All regurgitation cases under grade 2 in the description were categorized as grade 1.

Statistical analysis

Statistical analysis was performed using the IBM SPSS Statistics for Windows, Version 21 (Released 2012; IBM Corp., Armonk, New York, United States). The variables were investigated using visual (histograms, probability plots) and analytic methods (Kolmogorov-Smirnov/Shapiro-Wilk’s test) to determine whether or not they are normally distributed. Continuous variables were expressed as mean value ± standard deviation (SD). The chi-square test was employed to detect significant differences between categorical variables. A one-way analysis of variance (ANOVA) test with post-hoc analysis or Kruskal-Wallis test was used to determine statistically significant differences. The Mann-Whitney U test was performed to test the significance of pairwise differences using Bonferroni correction to adjust for multiple comparisons. Differences with a value of p < 0.05 were considered to be statistically significant.

## Results

A total of 31 elite athletes were included in this study, and their data were compared with those of age and sex-matched healthy 37 control subjects. The mean age of elite athletes was 24.3 ± 8.2 years, while that of the control group subjects was 25.5 ± 7.8 years (p = 0.48). All subjects in both the elite athlete and control groups were female. The demographic and clinical information of the elite athletes and control subjects is presented in Table 1. "No significant differences were noted in the demographic characteristics of elite athletes and healthy controls, except that the elite athletes were taller than the control group" (185.4 ± 7.4 vs. 162.5 ± 5.5, p = 0.01).

**Table 1:** Demographic characteristics and echocardiographic measurements of the professional athletes and controls D: diameter; LA: left atrium LV: left ventricle; EDD: end-diastolic diameter; ESD: end-systolic diameter; IVST: interventricular septum thickness; PWT: posterior wall thickness; RV: right ventricular; sPAP: estimated systolic pulmonary arterial pressure; EF: ejection fraction; E: transmitral peak E velocity; A: transmitral peak A velocity; e′: lateral mitral annulus early velocity; Vmax: maximal velocity A one-way ANOVA test with post-hoc analysis or Kruskal-Wallis test was used in order to determine statistically significant differences

Variables	Elite athletes (n = 31)	Healthy control group (n = 37)	p-value
Demographic characteristics of the professional athletes and controls			
Age (years)	24.3 ± 8.2	25.5 ± 7.8	0.48
Height (cm)	185.4 ± 7.4	162.5 ± 5.5	0.01
Body mass index (kg/m^2^ )	20.1 ± 2.4	21.1 ± 4.2	036
Smoking status (%)	3(9.6%)	5(13.5%)	0.24
Echocardiographic measurements of the professional athletes and controls			
LV EDD (mm)	48.0 ± 2.6	43.7 ± 2.5	0.04
LV ESD (mm)	31.1 ± 1.9	28.1 ± 2.2	0.09
IVST (mm)	8.5 ± 0.7	8.0 ± 0.6	0.24
PWT (mm)	8.7 ± 0.6	8.1 ± 0.7	0.46
Aort D (mm)	29.7 ± 2.4	27.5 ± 1.7	0.09
LA D (mm)	33.2±2.8	30.5±	0.55
Aortic Vmax (cm/sn)	1.19 ± 0.1	1.18 ± 0.1	0.06
RV EDD (mm)	24.5 ± 1.0	22.5 ± 1.3	0.49
LV EF (%)	62.2 ± 4.3	61.3 ± 4.2	0.41
Transmitral peak E velocity (cm/s)	0.95 ± 0.1	0.94 ± 0.1	0.23
Transmitral peak A velocity (cm/s)	0.64 ± 0.1	0.70 ± 0.1	0.47
Septal e′	12.1 ± 2.4	11,5 ± 3.1	0.25
E/A ratio	0.71 ± 0.1	0.72 ± 0.1	0.61
E/e’ ratio	5.2 ± 1.9	5.4 ± 2.1	0.41
sPAP (mmhg)	15.1 ± 6.3	11.0 ± 5.3	0.31

The 2D grayscale M-mode, color Doppler, and tissue Doppler transthoracic echocardiographic measurements are presented in Table 1. The left ventricular end-diastolic diameter was higher in elite athletes compared to healthy controls (48.0 ± 2.6 vs. 43.7 ± 2.5, p = 0.04); however, there were no statistically significant differences between elite athletes and healthy controls in the other echocardiographic measurements. The evaluation of the valves using grayscale M-mode and color Doppler was presented in Table 2. The elite athletes have more and higher degrees of mitral (p = 0.04) and tricuspid (0.003) valve insufficiencies compared to the control group. 

**Table 2:** Echocardiographic evaluations of valves using two-dimensional grayscale M-mode and color Doppler The Mann-Whitney U test was performed to test the significance of pairwise differences using Bonferroni correction to adjust for multiple comparisons

Grades	Elite athletes (n = 31)	Healthy control group (n = 37)	p-value
Mitral valve insufficiency			
Grade 0 (n)	15	25	0.04
Grade 1 (n)	5	8	
Grade 2 (n)	11	4	
Tricuspid valve insufficiency			
Grade 0 (n)	12	29	0.003
Grade 1 (n)	6	4	
Grade 2 (n)	13	4	
Pulmonary valve insufficiency			
Grade 0 (n)	25	34	0.36
Grade 1 (n)	3	1	
Grade 2 (n)	3	2	
Aortic valve insufficiency			
Grade 0 (n)	0	0	-
Grade 1 (n)	0	0	
Grade 2 (n)	0	0	

## Discussion

Athlete's heart can be defined as an abnormal but nonpathological heart. Various abnormalities encountered in the examinations of elite athletes can cause a confusing dilemma. Regurgitation of the heart valves is particularly common in elite female volleyball players. In our study, we observed a significant difference in mitral and tricuspid valve regurgitation among female volleyball players compared to the normal population. In addition, aortic valve leakage was not considered a component of the athlete's heart, and there was no significant difference in pulmonary valve regurgitation.

Exercise-induced cardiac remodeling is a physiological phenomenon in which the size, structure, mass, and function of the heart's chambers alter. In response to the increasing hemodynamic stress placed on the heart during repeated intense exercise sessions, this phenomenon represents an essential compensatory adaptation [8]. In our study, we observed that left ventricular end-diastolic diameters were larger in athletes. The average height of the athletes certainly contributed to this situation. However, pulmonary and aortic valves seem to be unaffected by these two parameters. During acute aerobic exercise in athletes, the increase in venous return significantly contributes to the rise in stroke volume, which subsequently increases left ventricular volume (i.e., preload). Wall stress increases as a result of the increase in preload and, consequently, chamber radius (i.e., end-diastolic volume), which in turn leads to a greater magnitude of contractility through the Frank-Starling mechanism. On the other hand, when preload declines and afterload increases during isometric exercises, stroke volume stays constant or even falls (although it is intensity dependent) [9]. One pivotal study found that systolic blood pressure (invasive brachial pressure) during a double leg press exceeded 320 mmHg [10]. Blood pressure increases during isometric/resistance exercise can be quite large and much exceed predicted levels [11]. It is evident that any modifications to the heart chambers' geometry could serve as a physiological leveling of the fluctuations in ventricular load.

Mitral and tricuspid valves may develop physiological coaptation abnormalities as a result of these modifications to the heart's structure [12]. In other words, it can be said that the structural changes in the athlete's heart primarily affect the atrioventricular valves. An indirect indicator of this physiological change would be the central valve regurgitation in the athlete's heart. Among evaluated athletes, all regurgitations were central leaks, and no eccentric leakage was observed. Considering the professionalism level of the study group and the fact that the majority of them were national team players, we can also comment that these valve regurgitations do not affect athletic performance. Importantly, the regurgitations observed were predominantly mild (grade 1) and did not correlate with any reported symptoms or decreased athletic performance among the participants. Nevertheless, the long-term clinical significance of such findings remains uncertain. While mild functional regurgitation is often considered benign in athletes, there is a need for longitudinal studies to determine whether these valvular changes persist, progress, or have any clinical implications over time.

Aortic valve leakage is not a finding we were expecting to see in female volleyball players. In addition, there was no difference in the ascending aorta dimensions compared to the normal population. The absence of significant changes in the pulmonary and aortic valves suggests that these valves may be less susceptible to exercise-induced remodeling, possibly due to their anatomical and functional characteristics. This observation is consistent with previous studies that have reported a higher prevalence of mitral and tricuspid regurgitation in athletes, with less frequent involvement of the aortic and pulmonary valves. One of the parameters in which no significant difference was observed was left ventricular wall thickness. A hypertrophic tendency in the left ventricle is typically expected in sports where resistance or strength-based loading predominates [13]. Interestingly, no evidence of cardiac hypertrophy was detected in our cohort. This may be attributed to the specific physical demands of volleyball, which is characterized by intermittent high-intensity activity with a relatively low static component. Such training may not provide the consistent pressure or volume overload necessary to induce significant cardiac hypertrophy, unlike endurance sports.

Given that our study focused exclusively on female athletes, it is important to consider sex-specific physiological factors that may influence cardiac adaptations. Prior research has indicated that women may exhibit different patterns of cardiac remodeling and valvular function compared to men, potentially due to hormonal influences and differences in body size and composition. These factors may contribute to the observed prevalence and characteristics of valvular regurgitation in our cohort [14].

Volleyball is one of the most popular Olympic sports worldwide. It is among the top female sports. However, there is limited literature on cardiac adaptations observed in female volleyball players. Our study was conducted with elite female volleyball players at the highest level and showed that left ventricular end-diastolic diameter, mitral valve insufficiency, and tricuspid valve insufficiency were more common in these athletes compared to the normal population. No difference was observed in other parameters, and if any other difference is detected during the examination, it should be approached with suspicion. This study uniquely contributes to sports physiology by providing detailed echocardiographic insights into valvular adaptations in elite female volleyball players, a demographic that has been underrepresented in existing literature. By identifying a higher prevalence of central mitral and tricuspid regurgitation without accompanying structural heart disease, the research enhances our understanding of physiological cardiac remodeling specific to this athletic population. These findings are instrumental in distinguishing between normal adaptive changes and potential pathological conditions, thereby refining clinical assessments and management strategies for female athletes.

Limitations

This study has several limitations. Firstly, the sample size was relatively small, comprising only 31 elite female volleyball players, which may limit the generalizability of the findings. Additionally, the cross-sectional design precludes assessment of longitudinal cardiac adaptations or potential progression of valvular changes over time. Echocardiographic evaluations were conducted at rest, without stress or exercise imaging, potentially overlooking dynamic valvular or functional alterations. Furthermore, the study did not account for intragroup variability, such as player positions, training intensity, or duration, which could influence cardiac remodeling. Lastly, the absence of advanced imaging modalities like cardiac magnetic resonance imaging (CMR) may have limited the precision in assessing right ventricular function and subtle myocardial changes.

## Conclusions

This study provides novel insights into the cardiac adaptations of elite female volleyball players, a group underrepresented in existing literature. Our findings indicate that over half of these athletes exhibit physiological central mitral and tricuspid regurgitation, typically not exceeding grade 2 in severity. These adaptations are believed to result from structural modifications in the heart due to intensive athletic training. Notably, no significant differences were observed in the prevalence of aortic or pulmonary valve insufficiency when compared to non-athletic individuals. These results contribute to a more nuanced understanding of the "athlete's heart" phenomenon, highlighting the need for sex-specific reference values in cardiac assessments. By focusing on female athletes, this study addresses a critical gap in sports cardiology and underscores the importance of considering gender differences in cardiac remodeling. Furthermore, our research emphasizes the necessity for tailored diagnostic criteria to accurately differentiate between physiological adaptations and potential pathological conditions in female athletes.
